# Dysfunctional autophagy following exposure to pro-inflammatory cytokines contributes to pancreatic β-cell apoptosis

**DOI:** 10.1038/s41419-017-0121-5

**Published:** 2018-01-24

**Authors:** Martine Lambelet, Leticia F. Terra, Makiko Fukaya, Kira Meyerovich, Leticia Labriola, Alessandra K. Cardozo, Florent Allagnat

**Affiliations:** 1 Department of Vascular Surgery, Centre Hospitalier Universitaire Vaudois (CHUV), Lausanne, Switzerland; 2 Departamento de Bioquimica, Instituto de Quimica, Universidade de Sao Paulo, Sao Paulo, Brazil; 3 ULB Center for Diabetes Research, Université Libre de Bruxelles, Brussels, Belgium

## Abstract

Type 1 diabetes (T1D) results from β-cell destruction due to concerted action of both innate and adaptive immune responses. Pro-inflammatory cytokines, such as interleukin-1β and interferon-γ, secreted by the immune cells invading islets of Langerhans, contribute to pancreatic β-cell death in T1D. Cytokine-induced endoplasmic reticulum (ER) stress plays a central role in β-cell demise. ER stress can modulate autophagic response; however, no study addressed the regulation of autophagy during the pathophysiology of T1D. In this study, we document that cytokines activate the AMPK-ULK-1 pathway while inhibiting mTORC1, which stimulates autophagy activity in an ER stress-dependent manner. On the other hand, time-course analysis of LC3-II accumulation in autophagosomes revealed that cytokines block the autophagy flux in an ER stress independent manner, leading to the formation of large dysfunctional autophagosomes and worsening of ER stress. Cytokines rapidly impair lysosome function, leading to lysosome membrane permeabilization, Cathepsin B leakage and lysosomal cell death. Blocking cathepsin activity partially protects against cytokine-induced or torin1-induced apoptosis, whereas blocking autophagy aggravates cytokine-induced CHOP overexpression and β-cell apoptosis. In conclusion, cytokines stimulate the early steps of autophagy while blocking the autophagic flux, which aggravate ER stress and trigger lysosomal cell death. Restoration of autophagy/lysosomal function may represent a novel strategy to improve β-cell resistance in the context of T1D.

## Introduction

The incidence of type 1 diabetes (T1D) is rising steadily in developed countries, with the recent, alarming prediction that it will double in children under the age of 5 by 2020^1^. Pancreatic β-cell depletion in T1D results from deregulated innate and adaptive immune responses. Pro-inflammatory cytokines (cyt) released by and/or expressed on the surface of the immune cells invading the islets contribute to β-cell apoptosis^2^. The interrelation of ER stress, inflammation, and mitochondrial dysfunction are major contributors to apoptosis in T1D^2^.

Autophagy is a catabolic process aimed at restoring energy homeostasis through self-digestion of intracellular proteins and organelles to survive under nutrient stress conditions. In addition, autophagy may alleviate the specific stress triggered by damaged organelles, such as the ER or mitochondria^3,4^. Autophagy plays a key role in maintaining pancreatic β-cell homeostasis and evidence is accumulating that autophagy protects β-cells against glucolipotoxicity and inflammation associated with T2D^5–11^. However, no study documented the putative role of autophagy in T1D. The aim of this study was to elucidate the regulation and contribution of autophagy to β-cell apoptosis in T1D. Our results confirm that autophagy is required for proper β-cell function and survival and shows for the first time that cyt impair the autophagy flux and trigger lysosomal cell death.

## Results

### Blocking autophagy rapidly and severely impairs rat β-cell viability

**Fig. 1:**
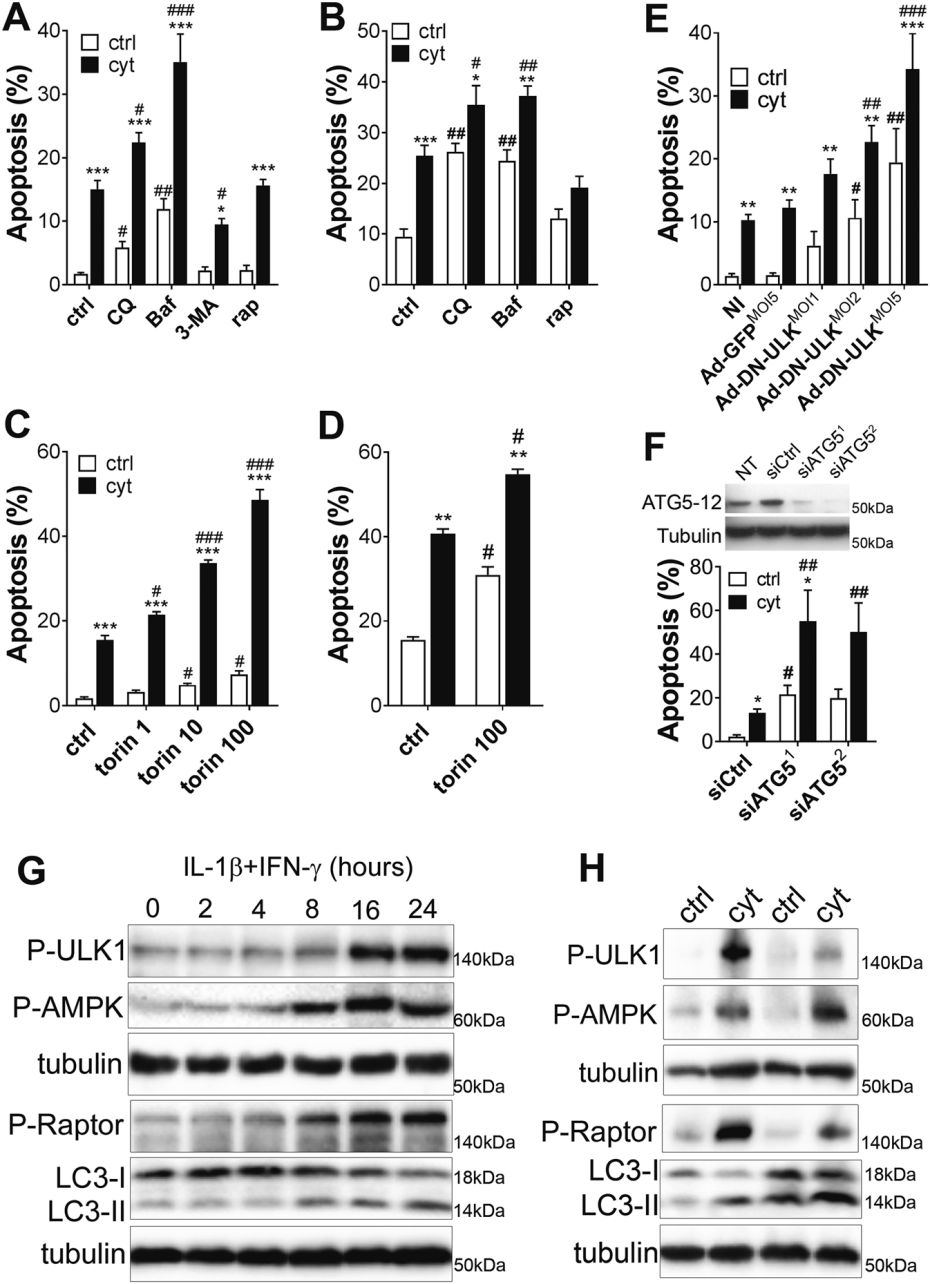
Pro-inflammatory cytokines stimulate the AMPK-ULK-1 axis while inhibiting mTORC1 in β-cells **a–d** Prevalence of apoptosis was evaluated by HO-PI staining in INS-1E cells **a, c** or primary rat islets **b, d** treated or not (ctrl) for 16 h with IL-1β + IFNγ (cyt), alone or in combination with chloroquine (CQ; 10 µM), Bafilomycin A1 (Baf; 100 nM), 3-Methyladenine (3-MA, 5mM), rapamycin (rap; 100 nM), or torin1 (1–100 nM, as indicated). Data are mean ± SEM of 4–6 independent experiments. *P < 0.05;**P < 0.01; ***P < 0.001 vs. ctrl. ^#^*P* < 0.05; ^##^*P* < 0.01 vs. IL-1β + IFNγ as determined by two-way ANOVA with Dunnet’s correction for multiple comparisons. **e** INS-1E cells infected with the Ad-DN-ULK-1 were treated or not (ctrl) with IL-1β + IFNγ (cyt) for 15 h. Prevalence of apoptosis was evaluated by HO-PI staining. Data are mean ± SEM of four independent experiments. **P < 0.01; ***P < 0.001 vs. respective ctrl condition (white bars). ^#^*P* < 0.05; ^##^*P* < 0.01 vs. respective Ad-GFP condition as determined by two-way ANOVA with post-hoc *t*-test with Sidak’s correction for multiple comparisons. **f** Upper panel*:* Western blot analysis of the ATG5–12 complex and tubulin in INS-1E cells transfected or not (NT) with a control siRNA (siCtrl) or two siRNA targeting ATG5 (siATG5^1 and 2^). Lower panel*:* prevalence of apoptosis in transfected INS-1E cells treated or not (ctrl) with cytokines for 24 h (cyt). Data are mean ± SEM of three independent experiments.*P < 0.05 vs. respective ctrl (white bars); ^#^*P* < 0.05, ^##^*P* < 0.01 vs. respective siCtrl condition as determined by two-way ANOVA with post-hoc *t*-test with Sidak’s correction for multiple comparisons. **g** Time-course Western blot analyses of P-AMPK, P-ULK-1, P-Raptor, LC3-I and II and tubulin in INS-1E cells treated with IL-1β + IFNγ. Data are representative of four independent experiments. **h** Western blot analysis of P-AMPK, P-Raptor, P-ULK-1, LC3-I and II and tubulin in primary rat islets exposed for 24 h to IL-1β + IFN-γ (cyt). Data are representative of four separate islet preparations

In order to investigate the involvement of autophagy in cytokine-induced pancreatic β-cell death we first tested the impact of autophagy inhibition on INS-1E cells and primary rat Langerhans islets viability in control conditions and after exposure to IL-1β and IFN-γ. Inhibition of autophagy activity through 16 h exposure to chloroquine (CQ; 10 µM) or bafilomycinA1 (Baf; 100 nM) decreased pancreatic cell viability (Fig. 1a, b), confirming that functional autophagy is required to β-cell survival^5,6^. A 16 h exposure to IL-1β + IFN-γ (cyt) in presence of those autophagy inhibitors further increased cell apoptosis, both in INS-1E cells and primary rat islet cells (Fig. 1a, b). Blocking autophagy initiation using the phosphatidylinositol 3-kinases (PI3K) inhibitor 3-Methyladenine (3-MA; 5 mM) reduced cytokine-induced apoptosis in INS-1E cells (Fig. 1a). Stimulating autophagy using the mTORC1 inhibitor rapamycin (rap; 100 nM) slightly protected rat islets, but not INS-1E cells, against cytokine-induced apoptosis (Fig. 1a, b). In contrast, stimulating autophagy using the very potent and selective mTOR inhibitor torin1 (1–100 nM) decreased basal viability and increased sensitivity to cyt, both in INS-1E cells and primary rat islets (Fig. 1c, d). Blocking autophagy in INS-1E cells using an adenoviral strategy to overexpress a dominant-negative form of the Unc-51-Like Kinase 1 (DN-ULK-1) (Fig. S1) confirmed that blocking autophagy induced INS-1E cell apoptosis and increased sensitivity to cyt at low multiplicity of infection (MOI), as assessed by Hoechst-PI staining (Fig. 1e) and cleaved caspase 3 Western blotting (Fig. S1). Similarly, blocking autophagy using siRNAs targeting ATG5 increased cytokine-induced apoptosis (Fig. 1f).

To better understand the effects of cyt and the various autophagy inhibitors on autophagy we studied the regulation of the AMPK-mTOR pathways, which regulate autophagy. A 16 h exposure to IL-1β + IFN-γ (cyt) stimulated AMPK phosphorylation in INS-1E (Fig. 1g) and rat islets (Fig. 1h). Downstream of AMPK, Raptor and ULK-1 were also phosphorylated, which correlated further down the pathway with dephosphorylation of mTOR targets eIF4E-binding protein 1 (4E-BP1) and 40S ribosomal protein S6 (S6RP) in INS-1E cells (Fig. S2). These data indicate that cyt stimulated AMPK while inhibiting mTORC1, which should drive increased autophagic activity. Time-course analysis of microtubule-associated protein 1 light chain 3 (LC3) in INS-1E cells revealed that cyt increased processing of LC3-I into LC3-II, the best marker of autophagosome formation to date^12^ (Fig. 1g). LC3-II levels were also increased after a 15 h treatment with cyt in rat islets (Fig. 1h). Immunocytochemistry experiments further suggested that cyt stimulated accumulation of autophagic vacuole in INS-1E cells as observed by LC3 and SQSTM1/p62 localization as punctae typical of autophagosome staining^12^ (Fig. S3A). Time-course analysis of p62 further revealed that cyt induced a progressive accumulation of p62 (Fig. S3B). The total levels of the Atg5-12 complex, which plays a key role in the regulation of autophagy, remained unchanged throughout the experiment despite the apparition of lower bands after 16/24 h of treatment, which may indicate degradation of the complex (Fig. S3C).

**Fig. 2:**
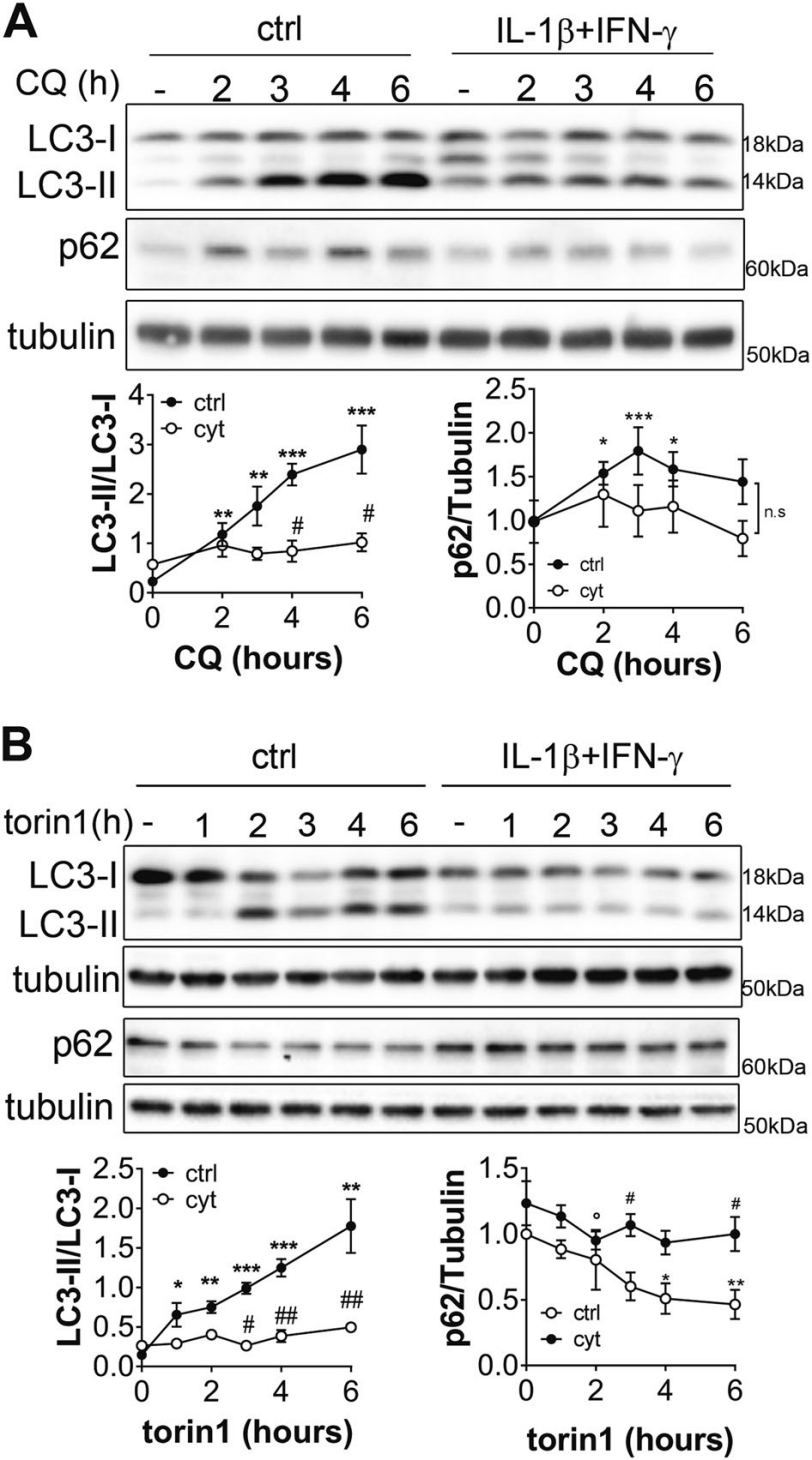
Time course analysis of LC3-II accumulation in INS-1E cells reveal that cytokines block autophagy flux Representative Western blot and quantification of LC3I/LC3II and p62/tubulin from INS-1E cells pre-treated or not (ctrl) with IL-1β + IFNγ for 6 h and treated with chloroquine (CQ) **a** or torin1 **b** for the indicated time. Data are mean ± SEM of 4–6 independent experiments. *P < 0.05, **P < 0.01, ***P < 0.001 vs. t0; ^#^*P* < 0.05 ^##^*P* < 0.01 vs. ctrl as determined by two-way ANOVA with ad-hoc post *t*-test with Sidak’s correction for multiple comparisons

LC3 accumulation can be observed in two situations: (i) autophagic activity is stimulated and (ii) autophagic flux is blocked and vesicles accumulate^12^. Time-course of autophagy blockade using CQ allows monitoring of the autophagy flux^12^. As expected, in control condition, autophagic flux blockade by CQ led to the accumulation of LC3-II and p62 due to the buildup of newly formed autophagosomes (Fig. 2a). In contrast, in cells pre-treated with cyt for 6 h, LC3-II and p62 levels did not significantly increase in presence of CQ (Fig. 2a), suggesting that the pre-treatment had already blocked the autophagic flux. Further experiments were performed using torin1 to stimulate autophagy^12^. In control condition, stimulating autophagy rapidly increased LC3-II levels, which reveals increased autophagosome formation, and decreased p62, which reveals autophagy-mediated protein degradation (Fig. 2b). The 6 h pre-treatment with cyt abolished the effect of torin1 on LC3-II and p62 levels (Fig. 2b), suggesting that the autophagic machinery was impaired by cytokine exposure.

**Fig. 3:**
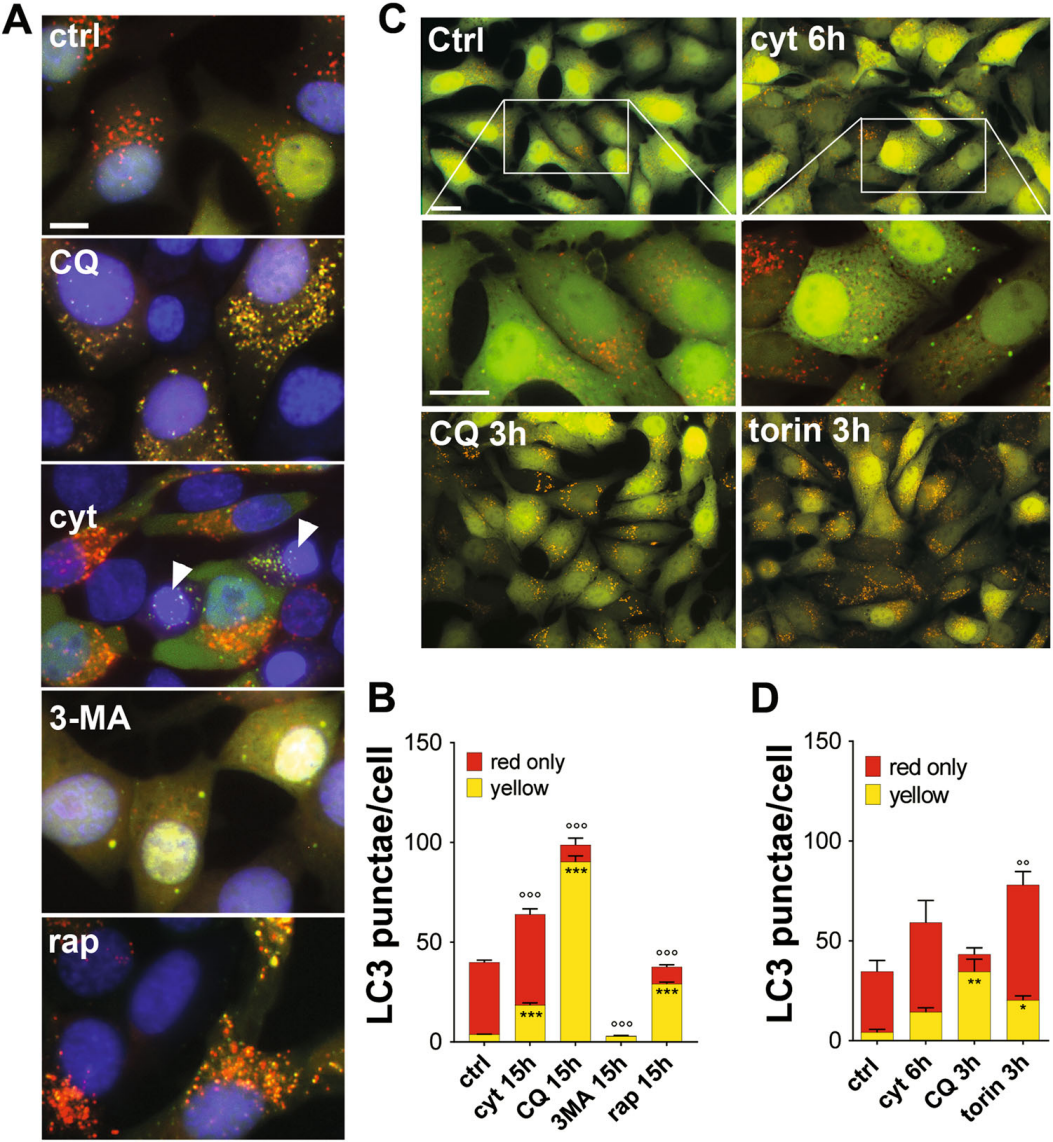
Cytokines trigger the accumulation of LC3-positive autophagosome in INS-1E cells **a, b** INS-1E cells infected with the Ad-mRFP-GFP-LC3 were treated with IL-1β + IFNγ, chloroquine (CQ), 3-methyladenine (3-MA), or rapamycin (rap) for 15 h. **a** Images are representative live-cell green-red overlay of LC3 fluorescence plus Hoechst nuclear staining (blue). Scale bar: 10 μm. Arrowhead indicate apoptotic nuclei. **b** Quantification of green and red (yellow) and red-only LC3 dots from **a**. Results are means ± SEM of four independent experiments. **c**, **d** INS-1E cells infected with the Ad-mRFP-GFP-LC3 were treated with IL-1β + IFNγ for 6 h, or chloroquine (CQ) or torin1 for 3 h. **c** Images are representative live-cell green-red overlay of LC3 fluorescence. Lower panels are two-fold magnifications of upper panels. Scale bar: 5 μm. **d** Quantification of green and red LC3 dots from **c**. Results are means ± SEM of six experiments. *P < 0.05, **P < 0.01, ***P < 0.01 vs. ctrl (yellow dots), °°*P* < 0.01, °°°*P* < 0.01 vs. ctrl (red dots), as determined by one-way ANOVA with Tukey’s correction for multiple comparisons

To further study the regulation of autophagic flux in β-cells, we generated an adenovirus (Ad-RFP/GFP-LC3) using the tandem fluorescent reporter plasmid mRFP-GFP-LC3 (kindly provided by Pr. T. Yoshimori). GFP fluorescence is quenched by the acidic pH environment of the lysosome, and is only visible as punctae in the early autophagosome stage, whereas mRFP exhibits more stable fluorescence in acidic compartments^13^. Thus, autophagosomes are labeled in yellow (mRFP and GFP signals; early stage) and autophagolysosomes are labeled in red (only mRFP signal; late stage). In control condition most of the RFP/GFP-LC3 staining was cytosolic with red/autophagolysosomes punctae and few yellow autophagosomes/LC3 punctae (Fig. 3), suggesting that autophagosomes have a short half-life compared to autophagolysosomes. As expected, in cells treated with CQ for 15 h, most of the cytosolic staining was replaced by accumulation of large yellow autophagosomes/LC3 punctae (Fig. 3a, b). After 15 h treatment with cyt, the number of yellow punctae increased by five-fold and the number of red punctae slightly increased (Fig. 3a, b). Of note, apoptotic cells display defective autophagy as evidenced by the accumulation of yellow-only punctae (Fig. 3a; cyt, arrowhead). We then studied LC3 fluorescence in cells treated with cyt for only 6 h, or cells treated with torin1 or CQ for 3 h (Fig. 3c, d), before the onset of apoptosis. As in the 15 h treatment, a 6 h treatment with cyt tended to increase the number of yellow punctae more than it increased the red-only punctae, although neither was actually significantly increased (Fig. 3c, d). The short 3 h treatment with torin1 increased both populations of punctae, with a more marked effect on yellow punctae, consistent with an increased number of newly formed autophagosomes. As expected, the short 3 h treatment with CQ treatment had a similar effect as the 15 h treatment (Fig. 3c, d).

**Fig. 4:**
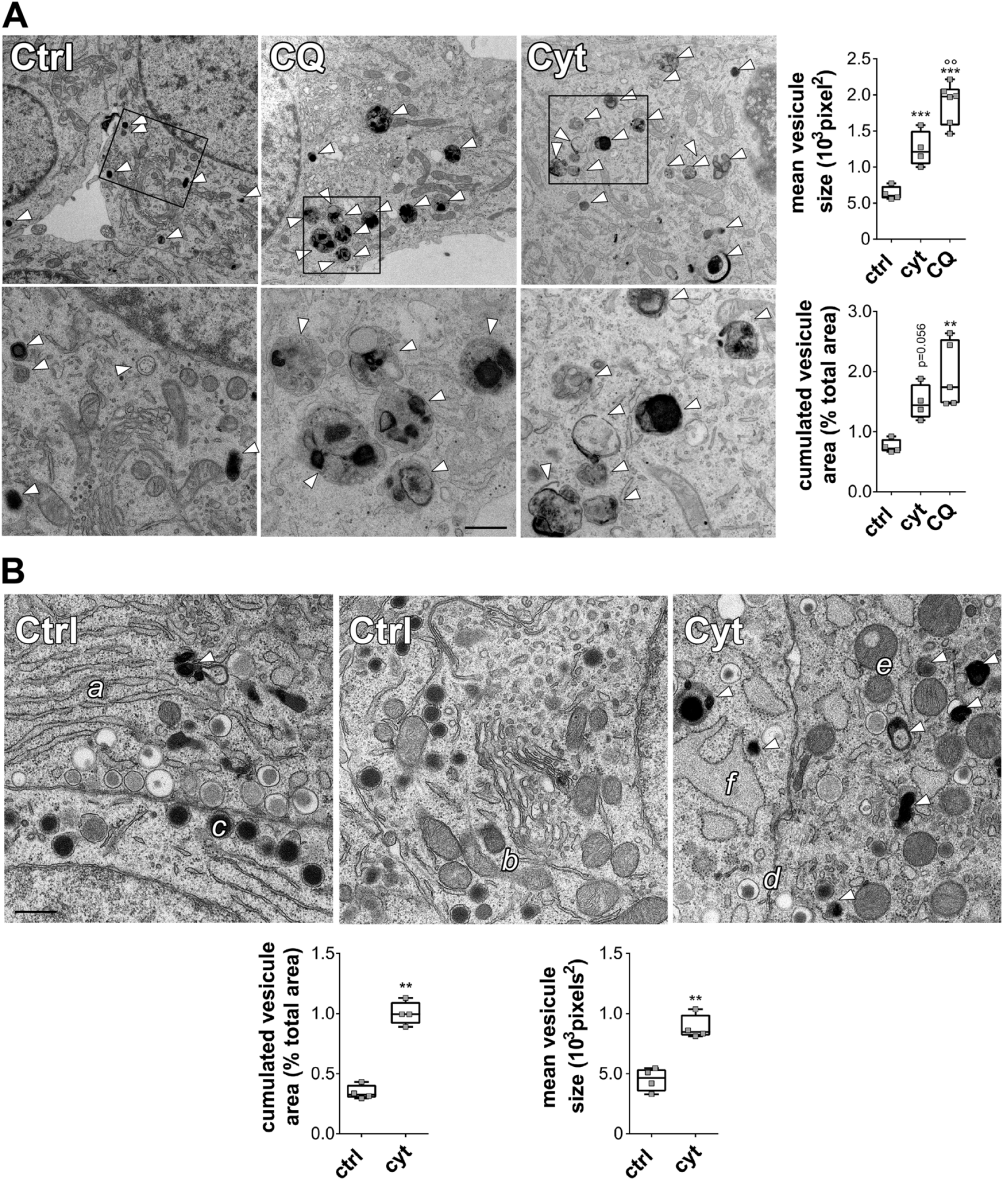
Cytokine (IL-1β + IFNγ) treatment rapidly leads to accumulation of multi-vesicular bodies with cargo in β-cells INS-1E cells **a** or primary rat islets **b** were treated with IL-1β + IFNγ for 8 h (**a**; INS-1E) or 12 h (**b**; rat islets), or chloroquine (CQ) for 5 h (**a**; INS-1E) before fixation and TEM imaging. **a** White arrowheads indicate electron dense autophagic vesicles. Lower panels are two-fold magnification of upper panels. **b** Letters indicate functional ER (**a**) and mitochondria networks (**b**), full insulin granules (**c**), insulin granules with low insulin content (**d**), fragmented mitochondrial network (**e**), and enlarged ER network (**f**). Representative images and quantification of electron dense autophagic vesicles shown as scatter plot with mean ± SEM of four independent experiments (*n* = 20 images per condition per experiment). **P < 0.01, ***P < 0.001 vs. ctrl; °°*P* < 0.01 vs. cyt as determined by one-way ANOVA with Tukey’s correction (**a**) or paired bilateral Student’s *t*-test (**b**). Scale bar 400 nm

Transmission electron microscopy (TEM) was then performed in INS-1E cells treated with cyt for 8 h or CQ for 5 h or in rat islets treated with cyt for 12 h. As compared to the control situation where autophagosomes were visualized as electron dense structures (Fig. 4a, arrowheads), the CQ treatment stimulated the formation of very large double membrane structures with cargo, suggesting the fusion of several autophagosomes (Fig. 4a). An 8 h exposure to cyt led to an intermediate phenotype between the control and CQ condition in INS-1E cells, i.e. accumulation of double membrane electron dense structures with cargo, albeit of smaller size and number (Fig. 4a). Similarly, in rat islets, a 12 h treatment with cyt resulted in the presence of larger double membrane electron dense structures covering a larger area of the rat β-cells (Fig. 4b). Of note, in primary rat β-cells, the area covered by autophagosomes is half of that in INS-1E cells, probably reflecting a higher autophagic activity in proliferating INS-1E cells than in primary β-cells. Altogether these data indicate impaired autophagic flux in cells treated with cyt, but not to the same extent as in CQ-treated cells. Paralleled with alterations in autophagosome size and number, the cytokine treatment also impaired the structure of the mitochondrial and ER network. Thus, in control conditions we observe functional ER (a) and mitochondria networks (b), as well as full insulin granules (c). In contrast, in cytokine-treated condition the insulin granules were lighter, which is typical of low insulin content (d), the mitochondria appeared rounder (e), and the ER network enlarged (f; Fig. 4b), which is consistent with mitochondrial swelling^11^ and ER stress^14^.

**Fig. 5:**
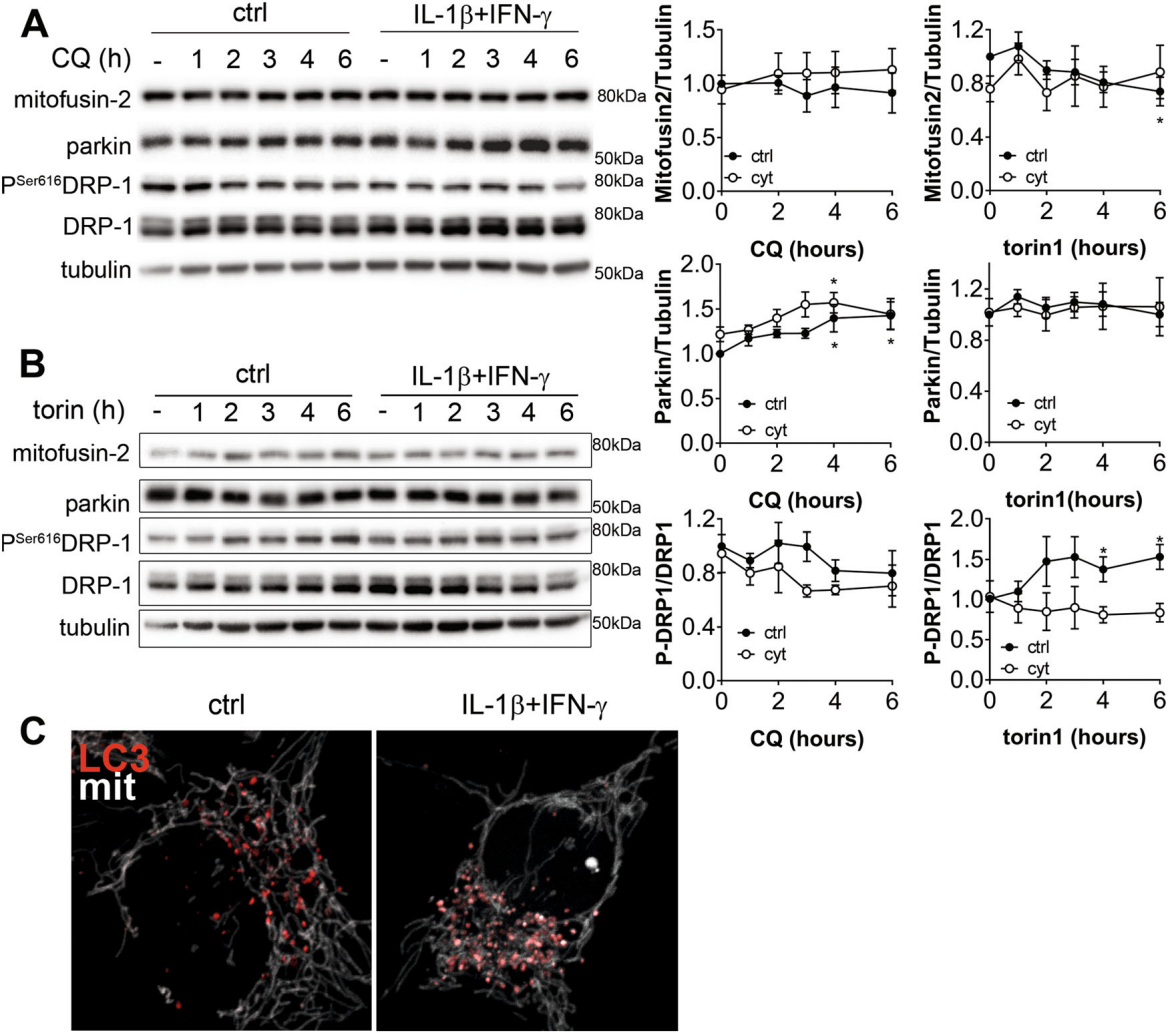
Cytokines have no major impact on mitophagy **a, b** Time-course Western blot analyses of chloroquine (CQ) **a** or torin1 **b** effect on Parkin, mitofusin2, P-DRP1, DRP-1, and tubulin levels in INS-1E cells pre-treated or not (ctrl) with IL-1β + IFNγ for 6 h. All quantifications are mean ± SEM of 4–5 independent experiments.*P < 0.05, **P < 0.01, ***P < 0.001 vs. t0; ^#^*P* < 0.05; ^##^*P* < 0.01; ^###^*P* < 0.001 vs. ctrl as determined by two-way ANOVA with ad-hoc post *t*-test with Sidak’s correction for multiple comparisons. **c** Live INS-1E cell imaging of Mitochondrial (white) and RFP-LC3 (red) in control (ctrl) and after a 6 h treatment with IL-1β + IFNγ. Images are representative of five independent experiments

ER stress and mitochondrial dysfunction are known events induced by exposure to pro-inflammatory cyt in β-cells^2^. Reticulophagy and mitophagy refer to macro-autophagy of the ER and mitochondria, respectively, to degrade dysfunctional part of the ER or mitochondrial network^15^. To assess mitophagy we again performed time-course CQ or torin1 exposure in INS-1E cells pre-treated or not (ctrl) with cyt for 6 h and studied the known mitophagy-associated proteins mitofusin2, parkin^16^, and P-Drp-1 (Fig. 5a, b). Mitofusin2 levels were not significantly modified by cyt or CQ, but reduced over time in response to torin1 treatment. Parkin levels increased with time in presence of CQ, but not in presence of torin1, and the cyt pre-treatment had no impact on parkin levels. P ^Ser616^DRP-1 levels tended to decrease in presence of CQ and significantly increased in presence of torin1. Additionally the cytokine pre-treatment did not impact the effect of CQ but blocked the effect of torin1. Altogether these data suggest that stimulating autophagy may impact mitochondrial fission/fusion, which maybe perturbed by cyt, although in a very subtle manner (Fig. 5a, b). INS-1E cells were then transfected with the mRFP-LC3 coding plasmid and plasmid expressing the pmTurquoise2 fluorescent protein specifically in the mitochondria^17^. In control condition there was little overlap between LC3 particles and the mitochondrial network. Upon a 15 h treatment with cyt the mitochondrial network was altered, presenting areas of fractionated swollen mitochondria. Moreover, the LC3 punctae seemed to co-localize more with the mitochondrial network than in control conditions (Fig. 5c).

**Fig. 6:**
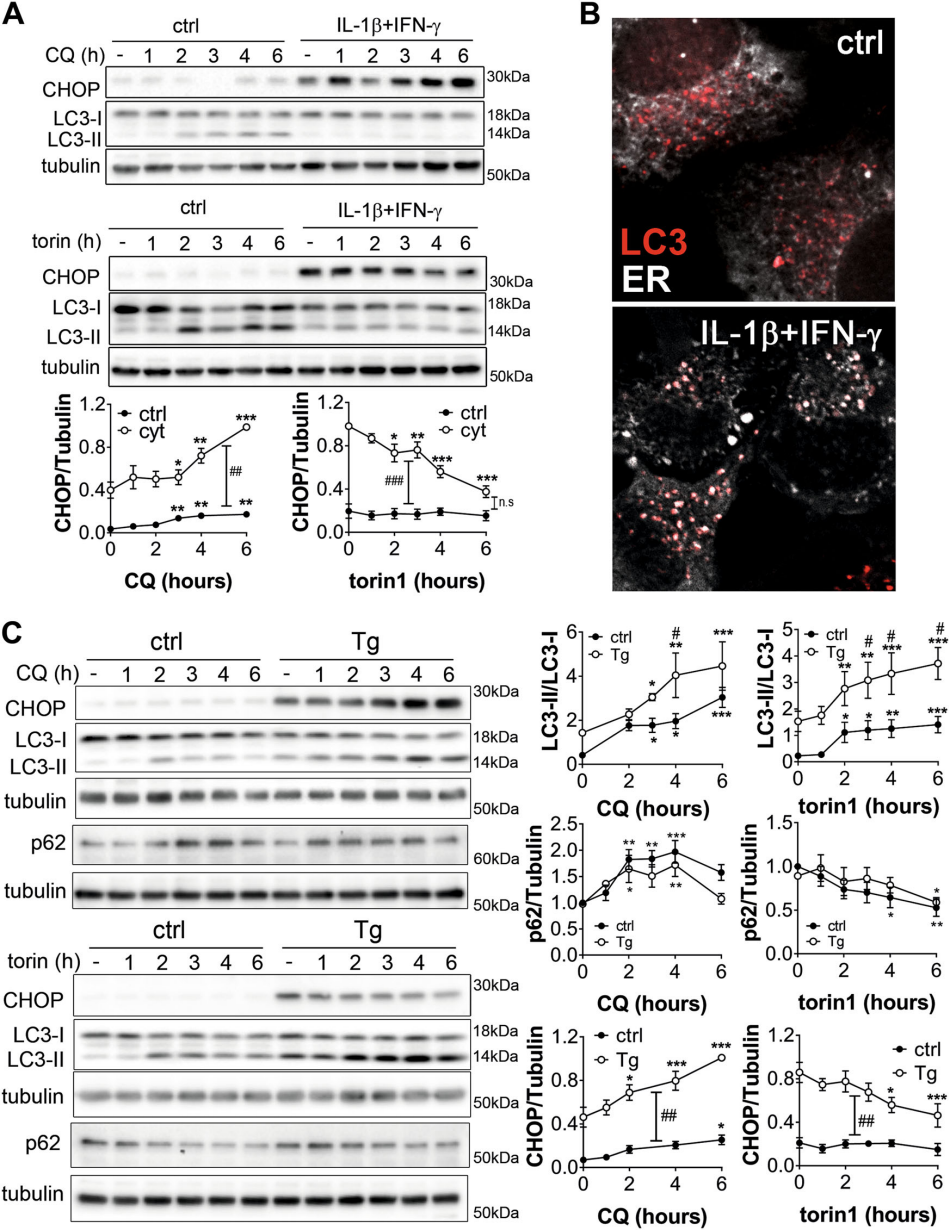
Blocking autophagy induces ER stress and CHOP expression but thapsigargin-induced ER stress does not increase autophagy flux **a** Time-course Western blot analyses of chloroquine (CQ) or torin1 effect on CHOP, LC3-I and II and tubulin levels in INS-1E cells pre-treated or not (ctrl) with IL-1β + IFNγ for 6 h. **b** Live INS-1E cell imaging of the ER network (white) and RFP-LC3 (red) in control (ctrl) and after a 6 h treatment with IL-1β + IFNγ. Images are representative of five independent experiments. **c** Time-course Western blot analyses of chloroquine (CQ) or torin1 effect on CHOP, p62, LC3-I and II and tubulin levels in INS-1E cells pre-treated or not (ctrl) with thapsigargin (Tg) for 4 h. All quantifications are mean ± SEM of 4–6 independent experiments. *P < 0.05, **P < 0.01, ***P < 0.001 vs. respective t0; ^#^*P* < 0.05; ^##^*P* < 0.01; ^###^*P* < 0.001 vs. respective ctrl as determined by two-way ANOVA with ad-hoc post *t*-test with Sidak’s correction for multiple comparisons

To assess ER stress and reticulophagy we studied the expression of the known ER stress marker CHOP. Both basal and cytokine-induced CHOP expressions were increased in presence of CQ, suggesting that blocking autophagy induced ER stress (Fig. 6a). Interestingly, torin1 exposure decreased cytokine-induced CHOP expression, indicating that increasing autophagic activity reduced ER stress (Fig. 6a).

INS-1E cells were then transiently transfected with a mRFP-LC3 coding plasmid and a plasmid expressing the pmTurquoise2 fluorescent protein specifically in the ER^17^. In control condition, LC3 particles slightly overlapped with the ER network. Upon a 15 h treatment with cyt large LC3 punctae co-localized with regions of bright condensed ER confirming major ER stress in those cells (Fig. 6b).

To further assess the role of ER stress in cytokine-induced dysfunction of autophagy, INS-1E cells were treated with the SERCA2 inhibitor thapsigargin (Tg). Time-course experiments of Tg exposure revealed that Tg strongly and rapidly stimulated phosphorylation of AMPK and ULK1, but not of Raptor, and stimulated the accumulation of LC3-II over LC3-I over time (Fig. S4A). In contrast, tunicamycin, which induces ER-stress through inhibition of glycosylation, had no effect on P-AMPK, P-Raptor, P-ULK-1 and LC3-I and II levels (Fig. S4B). Of note, downstream of mTOR, Tg treatment inhibited S6RP and 4EBP-1 phosphorylation in a time-dependant manner (Fig. S4A), similarly to cytokine treatment (Fig. S2), whereas increasing doses of tunicamycin inhibited S6RP phosphorylation but had no impact on P-4EBP-1 level (Fig. S4B). Thus ER calcium depletion, but not ER-stress per se, as induced by tunicamycin, stimulates the AMPK-ULK1 axis and autophagy in a similar way as cyt.

Further time-course experiments of CQ or torin1 exposure in INS-1E cells pre-treated or not (ctrl) with Tg for 4 h revealed that Tg increased autophagy activity but had no impact on the autophagy flux, since the kinetics of LC3-II and p62 modulations by the CQ or torin1 treatments were similar in control condition and in cells pre-treated with Tg (Fig. 6c). Of note, as in cells exposed to cyt (Fig. 6a), CQ or torin1 treatment, respectively, increased or decreased Tg-induced CHOP overexpression (Fig. 6c). Thus, it is likely that cytokine-induced autophagy dysfunction occurs independently of ER stress, although autophagy is likely very important to alleviate ER stress in β-cells.

The transcription factor EB (TFEB) is a master regulator of lysosome-based processes, including autophagy^18^, that controls the expression of many genes related to lysosomal function^19^. TFEB activation is mainly controlled through nuclear translocation. TFEB localization was studied in various conditions by Western blot of nuclear fractions and by live-cell imaging of INS-1E cells transfected with a GFP-TFEB construct. In control condition, TFEB was mostly cytosolic whereas upon treatment with torin1, TFEB rapidly translocated to the nucleus and accumulated at the surface of lysosomes. Similarly, CQ stimulated TFEB nuclear translocation (Fig. S5A–B). In contrast, Tg had no effect on TFEB localization. Interestingly, cyt triggered partial and heterogeneous TFEB translocation, suggesting complex effects of the cytokine treatment on lysosomal function and activity (Fig. S5A–B). Of note the transcript levels of several lysosomal genes controlled by TFEB^20^ were decreased upon exposure to cyt, confirming dysfunctional lysosome regulation in response to cyt despite partial translocation of TFEB (Fig. S5C).

**Fig. 7:**
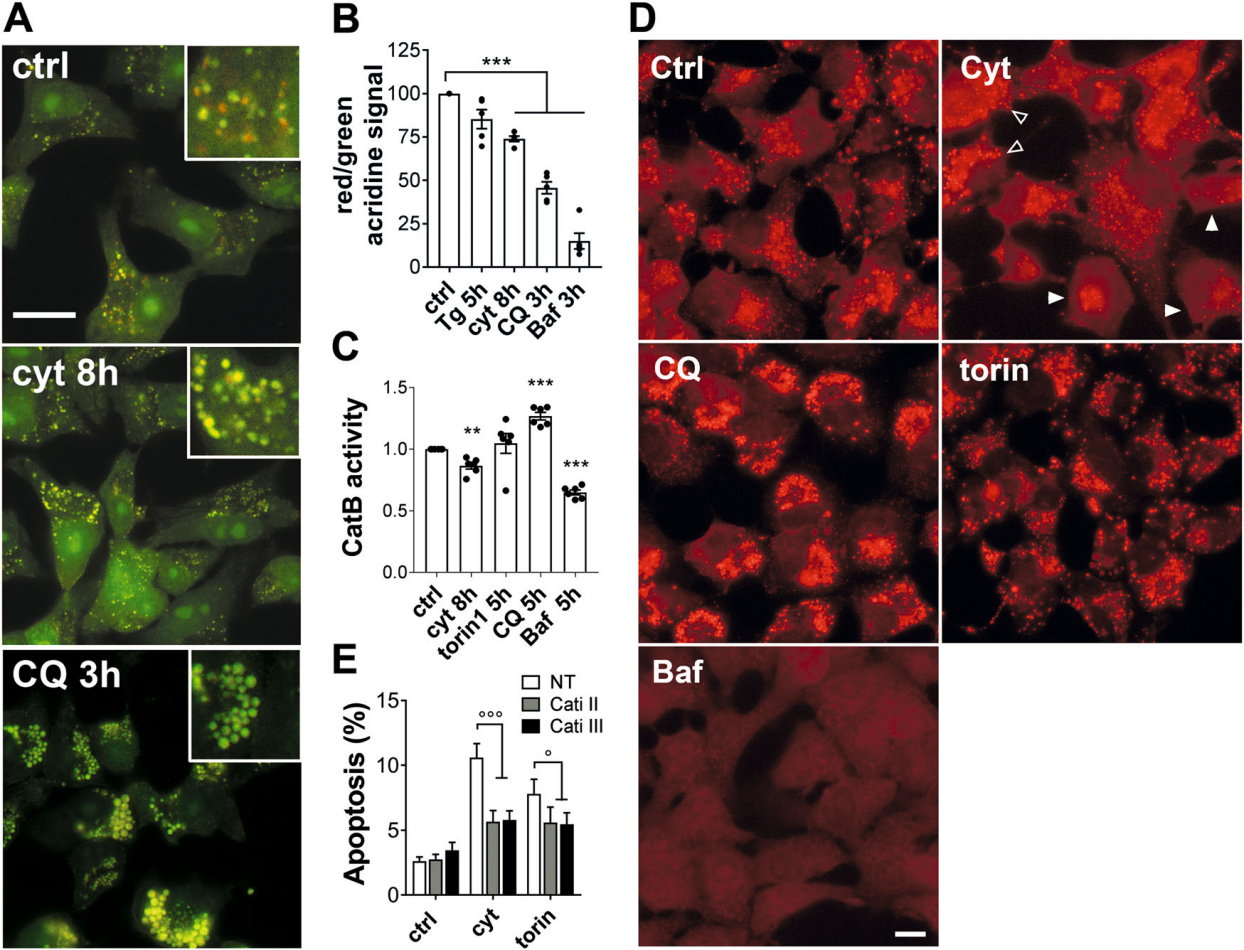
Cytokines induce lysosomal membrane permeabilization and Cathepsin B leakage **a, b** Acridine staining in INS-1E cells treated or not (ctrl) with IL-1β + IFNγ (cyt) for 8 h, chloroquine (CQ) or bafilomycin (Baf) or torin1 for 3 h, or thapsigargin (Tg) for 5 h. **a** Representative live INS-1E cell imaging of acridine staining (red + green signal overlay). Insets are three-fold magnifications. **b** Quantitative assessment of red (650 nm) over green (525 nm) acridine fluorescent signal as assessed in live cells using a fluorescent microplate reader. **c, d** Cathepsin B activity assay using cresyl violet fluorescence (628 nm) in live INS-1E cells treated or not (ctrl) with IL-1β + IFNγ (cyt) for 8 h, or chloroquine (CQ), bafilomycin (Baf), or torin1 for 3 h. **c** Fluorescence (628 nm) signal as assessed by mutiplate reader. **d** Representative images; full arrows point cells with cathepsin leakage, empty arrows point cells with accumulation of large dots of Cathepsin B activity; scale bar represents 10 µm. **e** Prevalence of apoptosis was evaluated by HO-PI staining in INS-1E cells treated or not (ctrl) for 16 h with IL-1β + IFNγ or torin1, alone or in combination with Cathepsin B Inhibitor II or Cathepsin Inhibitor III (Cati II or III; 10 µM). **P < 0.01, ***P < 0.001 vs. ctrl; °*P* < 0.05, °°°*P* < 0.001 vs. cyt as determined by one-way ANOVA with Tukey’s correction

To further assess lysosomal function we used Acridine Orange and a Cathepsin B enzyme activity assay. Acridine Orange is a dye accumulating in the acidic lysosomal compartment where it is protonated and sequestered, shifting its emission spectrum from green to red. INS-1E cells were treated or not (ctrl) with cyt for 8 h, Tg for 5 h or CQ or Baf for 3 h, then stained for 30 min with Acridine Orange and observed by immunofluorescence. As expected, in control condition, we observed a diffuse green cytosolic and nuclear monomeric form of acridine orange and red dots of protonated acridine aggregates sequestered in lysosomes (Fig. 7a). In contrast, in cells pre-treated with cyt the aggregates were bigger but there was a loss of red signal. Baf, which inhibits vacuolar H^+^-ATPase (V-ATPase), completely prevented the formation of acridine aggregates (Fig. 7a). Quantification of the ratio of red (650 nm) over green (525 nm) fluorescence further revealed that cyt, CQ or Baf treatment significantly shifted the acridine signal toward “green” (Fig. 7b). Further study of the activity of lysosomal enzyme Cathepsin B in similar conditions revealed that short-term treatment with cyt slightly decreased Cathepsin B activity, while CQ increased it (Fig. 7c). Further microscopic observation of the cells revealed events of Cathepsin B leakage upon an 8 h treatment with cyt (Fig. 7d, full arrowhead), while other cells displayed aggregates of Cathepsin B similar to what is observed upon a 5 h CQ treatment (Fig. 7d, empty arrowhead). Of note, a 5 h torin1 treatment increased the number, but not the size of aggregates, while a 5 h Bafilomycin treatment fully blocked Cathepsin B activity (Fig. 7d). Finally, inhibition of Cathepsin activity using Cathepsin B Inhibitor II or Cathepsin Inhibitor III (Calbiochem) reduced apoptosis induced by a 15 h treatment with cyt or torin1 (Fig. 7e), suggesting that lysosomal membrane permeabilization and release of cathepsins into the cytosol contribute to cytokine-induced and autophagy-induced apoptosis.

## Discussion

In the present study, we examined the relationship between cytokine-induced β-cell apoptosis, ER stress, and autophagy in rat pancreatic β-cells. We observed that pro-inflammatory cyt IL-1β and IFN-γ inhibit mTOR while stimulating the AMPK-ULK-1 axis to stimulate autophagy. However, these cyt also impair the lysosomal activity, thereby blocking autophagic flux, which aggravates ER stress and contributes to apoptosis.

In line with previous studies, we observed that autophagy is a pre-requisite to β-cell survival, supporting the hypothesis that β-cells strongly rely, due to their high protein production activity, on functional adaptive stress response to adjust to the ever changing insulin demand^21,22^. Thus, blocking autophagy by different means (CQ, bafilomycin or using a dominant negative ULK-1 or siRNAs targeting ATG5) induces β-cell apoptosis and potentiates cytokine-induced β-cell apoptosis. In contrast, inhibition of autophagy using 3-MA reduces cytokine-induced apoptosis. However, 3-MA may act through autophagy-independent pathways, such as inhibition of class I PI3K^23,24^. In nutrient rich conditions, such as ours, 3MA may even stimulate autophagy flux instead of blocking it^23^. In addition, a study in Hela and HEK293 cells reported that 3MA may inhibit the UPR^25^, which would protect β-cells against cytokine-induced apoptosis. Further studies are required to elucidate how 3-MA protects against cyt and ascertain whether or not its effects are linked to autophagy. Interestingly, rapamycin treatment slightly reduces cytokine-induced apoptosis in rat islets, but not in INS-1E cells. However, rapamycin poorly stimulates autophagy in mammalian cells and its effect on rat primary islets might not be related to autophagy^26^. In contrast the very potent autophagy inducer torin1 was cytotoxic, in line with reports that excessive autophagy may induce cell death^27^.

Although cyt stimulate the AMPK-ULK-1 axis, they block the autophagic flux through a distinct pathway. Thus, cyt induce the AMPK-ULK-1 axis in an ER stress-dependent manner. Actually, ER stress per se, as induced by tunicamycin, does not stimulate the AMPK-ULK-1 pathway and LC3-II accumulation. It is ER Ca^2+^ depletion, as induced by Tg, which is sufficient to stimulate the AMPK-ULK-1 axis and the formation of new autophagosomes. However, ER Ca^2+^ depletion has no impact on the autophagic flux, as assessed by time-course of LC3-II accumulation in presence of Tg and CQ. From these findings we concluded that cyt activate autophagy via ER Ca^2+^ release and ER stress. On the other hand time-course analyses of LC3-II and p62 protein levels in presence of CQ or torin1 in cells pre-treated with cyt clearly demonstrated that cyt impair the autophagic flux. Given that Tg has no effect on the autophagic flux cyt likely impair autophagic flux independently of ER Ca^2+^ release and ER stress.

Live-cell microscopy and TEM imaging further show that cyt trigger the accumulation of large GFP-LC3-positive double-membrane bodies in β-cells, suggesting impaired autophagolysosome function, but no defect in the fusion of autophagosomes and lysosomes. The lack of GFP fluorescence quenching in cytokine-treated condition suggests impaired acid environment in the lysosome. This hypothesis is supported by acridine staining experiments, which indicate increased lysosomal pH upon cytokine exposure. Further study of Cathepsin B activity confirmed that cyt induce lysosome dysfunction and Cathepsin B imagery revealed signs of cathepsin leakage, a feature of lysosomal membrane permeabilization that initiates the lysosomal apoptosis pathway^28,29^. Cathepsin inhibition partly protects against cytokine-induced apoptosis, indicating that cathepsin release contributes to cytokine-induced apoptosis. In line with a key role of cathepsin release, it was previously shown that Bid contributes to cytokine-induced apoptosis^30^, and Bid truncation by cathepsin play a key role in the lysosomal apoptosis pathway^28,29^. Interestingly, a recent study showed that GLP-1 may protect β-cells against palmitate-induced apoptosis by preventing lysosomal membrane permeabilization and restoring autophagic flux^31^. Altogether these evidences support the concept that blockade of autophagic flux is the result of lysosomal dysfunction and cathepsin leakage, which contribute to cytokine-induced apoptosis. Several external factors may induce lysosomal membrane permeabilization^28^ and further studies are required to better understand how cyt induce lysosomal membrane permeabilization to initiate the lysosomal apoptosis pathway. Upstream lysosomal membrane permeabilization we propose defective nuclear translocation of TFEB as a possible explanation for cyt-induced lysosomal dysfunction. TFEB is a master regulator of autophagy machinery and overexpression of its target genes supports autophagy flux^19^. Thus, the defective TFEB activation we observed in response to cyt may contribute to cytokine-induced lysosomal dysfunction.

In this study, we also demonstrate that blocking autophagy using CQ stimulates the expression of the ER stress marker CHOP and aggravates cytokine-induced or Tg-induced CHOP overexpression and apoptosis. This confirms previous reports showing that blocking autophagy results in ER stress in β-cells^32–35^. Live-cell imaging of mitochondrial and ER networks further suggests that autophagy of the ER (reticulophagy) is probably important in the context of cytokine exposure, as LC3 accumulates in regions of distended ER. Thus, the blockade of autophagy flux aggravates cytokine-induced ER stress and induces a vicious circle of ER dysfunction that cannot be resolved in absence of functional autophagy.

**Fig. 8:**
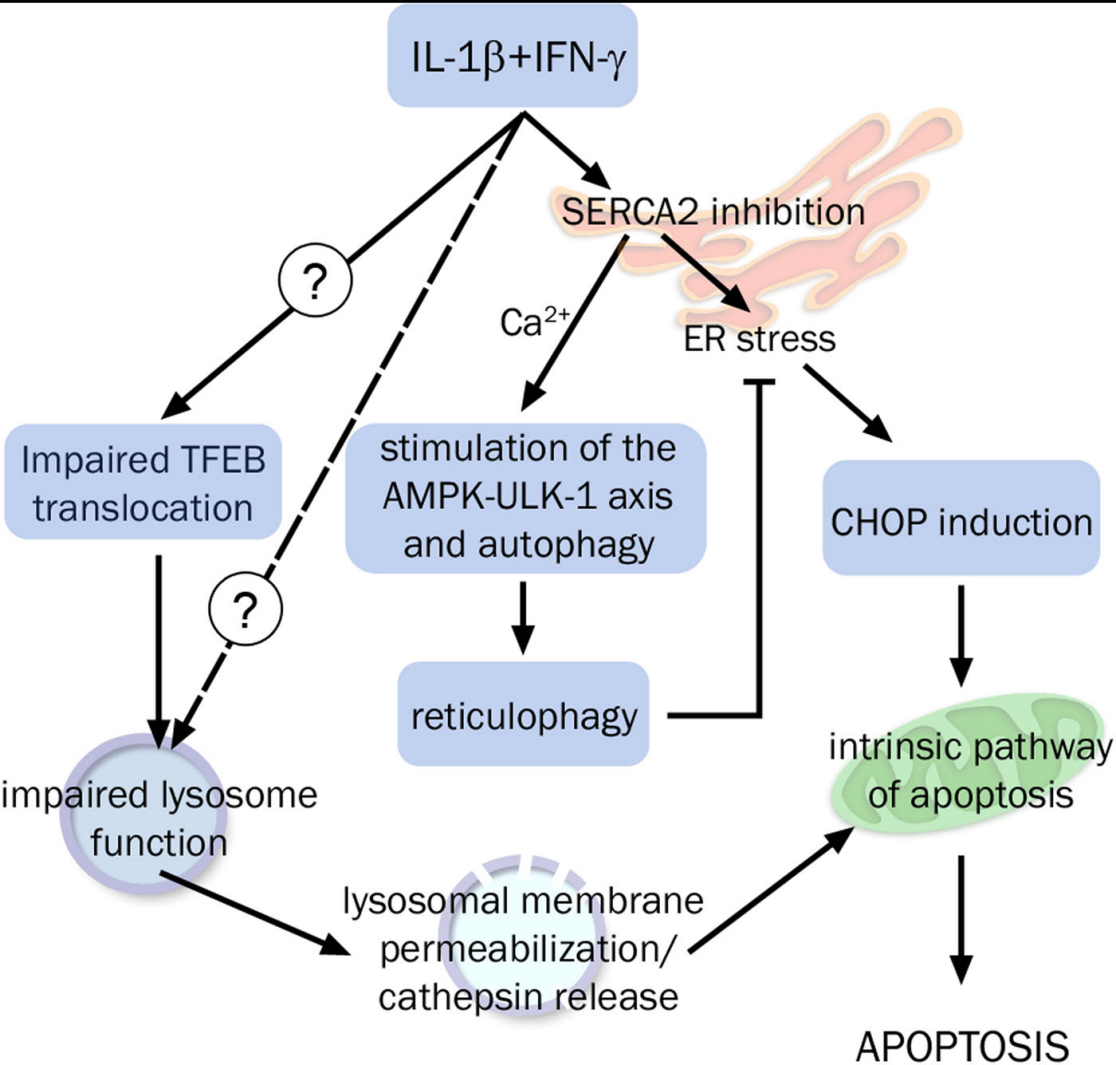
Cytokine-mediated blockade of autophagy sensitize β-cells to ER stress-induced apoptosis Pro-inflammatory cytokines trigger ER stress and increased CHOP expression. The ER stress-mediated [Ca^2+^]_i_ increase promotes AMPK phosphorylation, inhibition of mTORC1, and the subsequent activation of ULK-1 and autophagy. Autophagy may alleviate ER stress. However, cytokines induce lysosomal membrane permeabilization and cathepsin leakage, which contributes to the intrinsic pathway of apoptosis. Autophagy blockade aggravates cytokine-induced ER stress, CHOP overexpression, and eventually apoptosis

In conclusion we propose the following: cyt rapidly induce lysosome dysfunction, leading to dysfunctional autophagy, which aggravates ER stress. ER stress and cathepsin release converge to trigger apoptosis via the mitochondrial pathway. Autophagy and ER stress are intertwined in the adaptive stress response triggered by cyt. Thus, while cytokine-induced ER stress stimulates autophagy, cyt inhibit lysosomal function and autophagy flux, which promotes ER stress and trigger the lysosomal apoptosis pathway (Fig. 8).

Many studies suggest that the number of autophagosomes and the autophagic flux are increased in several type 2 diabetes models^6,11,36^, as well as in islets from type 2 diabetic patients^10,37^. Similarly, in vitro it was shown that free fatty acids stimulates autophagic flux and enhances the formation of autophagosomes and autolysosomes in β-cells^9,38–41^. However, recent in vitro and in vivo evidences suggest that long-term exposure to free fatty acids suppresses autophagy in β-cells^42^ and that the increase in autophagosome machinery in fatty acid-induced pancreatic failure may actually reflect an impairment of autophagic flux^10,31,43^. Thus, it could be that the autophagy flux is impaired both in type 1 and type 2 diabetes. Further assessment of autophagy flux in β-cells is required to determine whether autophagic dysfunction occurs in the β-cells of patients with both types of diabetes. Understanding the regulation of autophagy flux is of paramount importance for therapeutic purposes as it would be preferable to restore autophagy and lysosome function, rather than attempting to stimulate dysfunctional autophagy, which exacerbates ER stress and the susceptibility of β-cells to apoptosis.

## Research design and methods

### Materials

The following chemicals were dissolved in DMSO and used as indicated: A23187 (Sigma; C7522; 1 μM), Tg (Tocris Bioscience; 1138; 100 nM), tunicamycin (Sigma; T7765; 5 µg/ml), torin1 (Tocris Bioscience; 4247; 1–100 nM), Bafilomycin A1 (Sigma; B1793; 100 nM), rapamycin (Calbiochem | 553210—Merck Millipore; 100 nM). Cathepsin inhibitor III (Calbiochem | 219419—Merck Millipore; 10 μM). CQ (Sigma; C6628; 20 μM) and Cathepsin inhibitor II (Calbiochem | 219385—EMD Millipore; 10 μM) and Autophagy Inhibitor 3-MA (Calbiochem | 189490—Merck Millipore; 5 mM) were dissolved in distilled water. The following cyt were used: recombinant human IL-1β (R&D systems, Abingdon, UK) at 10 U/ml in experiments with INS-1E cells and 50 U/ml in experiments with rodent islets; recombinant rat IFN-γ (R&D systems, Abingdon, UK) at 100 U/ml (7.2 ng/ml) in experiments with INS-1E cells and 500 U/ml (36 ng/ml) in experiments with rat primary islets^44,45^. The concentrations of chemicals and cyt were selected based on our previous dose–response studies^45^.

### Cells and islets

The rat insulinoma cell line INS-1E (kindly provided by Prof. Pierre Maechler, CMU, University of Geneva^46^) was maintained in complete RPMI 1640 medium, as previously described^44,47^. Male Wistar rats (Janvier, France) islets were isolated by collagenase digestion followed by hand picking under a stereomicroscope^44,47^. Rat care, surgery, and euthanasia procedures were approved by the ethical committee of the Centre Hospitalier Universitaire Vaudois and the Cantonal Veterinary Office (Service de la Consommation et des Affaires Vétérinaires SCAV-EXPANIM, N° VD2543).

### RNA isolation and quantitative RT-PCR

Cells were homogenized in Tripure Isolation Reagent (Roche Diagnostics AG, Rotkreuz, Switzerland), and total RNA was extracted using the kit procedure. Transcripts (1 μg) were reverse-transcribed using ImProm-II Reverse transcription System (Catalys AG, Wallisellen, Switzerland). Quantitative PCR was performed in a StepOne plus apparatus, using the Applied Biosystems Fast SYBR^©^ Green Master mix (Life Technologies Corporation, Carlsbad, CA, USA). The primers used for amplification are given in Table S1. Expression values were normalized to the ribosomal protein L27 (Rpl27).

### Adenoviral constructs

Ad-DN-ULK1 was generated based on a plasmid encoding the a mutant ULK1 (K46I) that works as a dominant-negative of ULK1/2 activity and inhibits autophagy^48^ (kindly provided by Dr. Sharon Tooze (London Research Institute)). Ad-R/GFP-LC3 (Ad-LC3) was generated from a tandem fluorescent reporter plasmid mRFP-GFP-LC3 (Addgene plasmid #21074; kindly provided by Pr. T. Yoshimori)^13^. All viral vectors were generated and purified by Vector Biolabs, Philadelphia, PA, USA. Adenoviral infections were conducted as previously described^44,49,50^.

### Live-cell imaging of tagged fluorescent proteins

Mitochondrial or ER staining was achieved by transient transfection of plasmids pmTurquoise2-Mito (Addgene plasmid #36208) or pmTurquoise2-ER (Addgene plasmid #36204) (gift from Dorus Gadella)^17^. These were co-transfected with plasmid mRFP-LC3 (Addgene plasmid #21074; kindly provided by Pr. T. Yoshimori)^13^.

TFEB imaging was achieved by transient transfection of plasmids pEGFP-N1-TFEB (gift from Shawn Ferguson) (Addgene plasmid # 38119)^51^. Quantitative assessment of the TFEB nuclear localization was achieved by dividing the density of the nuclear signal by the mean density of the fluorescent signal in each cell using the ImageJ software. Fifteen images per condition from five distinct experiments were assessed.

Ad-LC3 imaging was achieved 48 h post infection (MOI1) on INS-1E cells plated on glass cell culture chamber (Ibidi) and photographed on a Leica inverted microscope (×40 oil immersion fluorescence objective). The Ad-LC3 green and red stainings were first converted to a 16 bit format and the signal to noise ratio was determined by applying the Yen threshold method^52^. A binary image was then created and the number of particles (LC3 dots) was measured. Data were normalized to the number of cells in each image.

### Acridine orange staining

INS-1E cells in 24-well plates were loaded for 30 min at 37 °C, 5% CO_2_ in the dark in KRBH (130 mM NaCl, 4.8 mM KCl, 0.5 mM NaH_2_PO_4_, 5 mM NaHCO_3_, 2 mM CaCl_2_, 1.2 mM MgCl_2_, 10 mM HEPES, 1 mM CaCl_2_) as previously described^44^ supplemented with 1 µM Acridine Orange (Enzo Life Science). Following loading, cells were washed in KRBH and fluorescence was immediately quantified using the Synergy Mx Multi-Mode Microplate Reader (BioTek AG, Luzern, Switzerland) with filter sets for green (Ex = 485 nm, Em = 525 nm) and red (Ex = 460 nm, Em = 650 nm) fluorescence. Fluorescent signal was then expressed as the ratio of “red” over “green” signal. Alternatively, cell were plated on glass cell culture chamber (ibidi GmbH; Martinsried, Germany) and photographed on a Leica inverted microscope with green and red fluorescence filter (×40 oil immersion objective).

### Cathepsin B activity

Cathepsin B activity was measured using Enzo Life Science’s CV-Cathepsin B Detection Kit according to manufacturer’s instruction (Enzo Life Science). The kit utilizes the non-cytotoxic cell permeant, photostable fluorophore, cresyl violet (CV), which generate red fluorescence when excited at 550–590 nm upon cleavage by cathepsin. Briefly, INS-1E cells in 24-well plates were loaded for 30 min at 37 °C, 5% CO_2_ in the dark in KRBH supplemented with the substrate CV-(RR)2 (CatB). Following loading, cells were washed in KRBH and fluorescence was immediately quantified using the Synergy Mx Microplate Reader (BioTek AG, Luzern, Switzerland) with filter sets (Ex = 592, Em = 628 nm) and the data were normalized to control (ctrl). For live cell imaging INS-1E cells were plated on glass cell culture chamber (ibidi) and photographed on a Leica inverted microscope with red fluorescence filter (×40 oil immersion objective).

### Immunofluorescence

INS-1E cells grown on glass coverslips were fixed for 5 min in 4% ice-cold paraformaldehyde, rinsed in phosphate buffered saline (PBS) and permeabilized for 30 min in PBS supplemented with 1.5% BSA and 0.2% Triton X-100. Coverslips were incubated overnight at 4 °C in the presence of antibody against LC3B and p62 (dilution 1/100; see Table S2). Cells were then washed and further exposed for 1 h to an appropriate Alexa fluor 488-conjugated or 555-conjugated antibodies (1/1000; N.V. Life Technology AG, Switzerland), and photographed under fluorescence microscopy (Leica Camera AG, Nidau, Switzerland).

### Transmitted electron microscopy

INS-1E cells or islets of Langerhans were fixed during 2 h at room temperature (RT) in glutaraldehyde solution (EMS, Hatfield, PA, USA) 2.5% in phosphate buffer (PB 0.1 M pH7.4) (Sigma, St. Louis, MO, USA). After washing three times in PB buffer, samples were postfixed by a fresh mixture of osmium tetroxide 1% (EMS, Hatfield, PA, USA) with 1.5% of potassium ferrocyanide (Sigma, St. Louis, MO, USA) in PB buffer for 2 h at RT, washed three times in distilled water and dehydrated in acetone solution (Sigma, St. Louis, MO, USA). This was followed by infiltration in Epon (Sigma, St. Louis, MO, US) at graded concentrations (Epon 1/3 acetone-1 h; Epon 3/1 acetone-1 h, Epon 1/1–2 h; Epon 1/1–12 h) and finally polymerized for 48 h at 60 °C in oven. Ultrathin 50 nm sections were cut on a Leica Ultracut (Leica Mikrosysteme GmbH, Vienna, Austria) and picked up on a copper slot grid 2 × 1 mm (EMS, Hatfield, PA, USA) coated with a polystyrene film (Sigma, St. Louis, MO, USA). Sections were poststained with 4% uranyl acetate (Sigma, St. Louis, MO, USA) for 10 min, rinsed several times with water followed by Reynolds lead citrate during 10 min and rinsed several times with water. Micrographs were taken with a transmission electron microscope FEI CM100 (FEI, Eindhoven, The Netherlands) at an acceleration voltage of 80 kV with a TVIPS TemCam-F416 digital camera (TVIPS GmbH, Gauting, Germany). Positive signals were evaluated automatically using the ImageJ software on 20 images per experiment per condition. Sixteen-bit images were used and a thresholding method was used to select electron-dense particles. The selected staining was processed into a binary image and the number and size of particles were measured^53,54^.

### Western blot analysis

Cells were washed once with cold PBS and immediately lysed in Laemmli buffer as previously described^44,47^. Lysates were resolved by SDS-PAGE and transferred to a PVDF membrane. Immunoblot analyses were performed as previously described^44,47^ using the antibodies listed in Table S1.

### Assessment of cell viability

The percentage of viable, apoptotic, and necrotic cells was determined using the DNA-binding dyes Propidium Iodide (PI, 5 µg/ml) and Hoechst 33342 (HO, 5 µg/ml, Sigma-Aldrich)^45^. The cells were examined by inverted fluorescence microscopy (Leica Camera AG, Nidau, Switzerland). A minimum of 500 cells was counted in each experimental condition by two independent observers, one of them unaware of the sample identity.

### Statistical analysis

Data are presented as mean ± SEM. Comparisons were performed by two-tailed paired Student’s *t*-test or by one or two-way ANOVA followed by *t*-tests with Tukey’s correction for multiple comparisons. A *p*-value ≤ 0.05 was considered statistically significant.

## Electronic supplementary material

Supplemental Figure legends

Fig S1

Fig S2

Fig S3

Fig S4

Fig S5

supplemental tables
