# Supplementary material for: Dysfunctional autophagy following exposure to pro-inflammatory cytokines contributes to pancreatic β-cell apoptosis
Source: Cell Death Dis. 2018 Jan 24;9(2):96. doi: 10.1038/s41419-017-0121-5 (PMC5833699; doi:10.1038/s41419-017-0121-5)
Supplement: Supplementary file 1 — Supplemental Figure legends [file 41419_2017_121_MOESM1_ESM.docx]

**Supplemental Figure legends**

**Figure S1**

Western blot analysis of ULK, cleaved caspase 3 and tubulin in INS-1E cells infected with the Ad-DN-ULK-1 were treated or not (ctrl) with IL-1β+IFNγ (cyt) for 15h. *Left panel*: representative Western Blot; *right panel*: data are mean±SEM of 5 independent experiments *P<0.05; **P<0.01; ***P<0.001 vs. respective ctrl (white bar) condition. #P<0.05; ##P<0.01; ###P<0.001 vs. respective GFP-infected condition as determined by one-way ANOVA with post-hoc t-test with Sidak’s correction for multiple comparisons.

**Figure S2**

Time-course analyses of P-AMPK, AMPK, P-S6RP, S6RP, P-4E-BP1, 4E-BP1 and tubulin in INS-1E cells treated with IL-1β+IFNγ, as indicated. This figure is representative of 4 independent experiments.

**Figure S3**

LC3 (green) and p62 (red) immunolabeling in INS-1E cells treated or not (ctrl) with IL-1β+IFNγ for 15h. Scale bar: 5μm. Images are representative of 4 distinct experiments. **B-C**) Western blot analysis of p62 (**B**) and the ATG5-12 complex (**C**) in INS-1E cells exposed to IL-1β+IFNγ for the indicated time. *P<0.05; ***P<0.001 vs. t0 as determined by one-way ANOVA with post-hoc t-test with Tukey’s correction for multiple comparisons.

**Figure S4**

**A**) Time-course analyses of P-AMPK, AMPK, P-Raptor, LC3, P-ULK1, LC3, P-S6RP, S6RP, P-4E-BP1, 4E-BP1 and tubulin in INS-1E cells treated with thapsigargin. **B**) Dose-response analyses of P-AMPK, AMPK, P-Raptor, LC3, P-ULK1, LC3, P-S6RP, S6RP, P-4E-BP1, 4E-BP1 and tubulin in INS-1E cells treated with tunicamycin, as indicated. Blots are representative of 4 independent experiments.

**Figure S5**

**A-C**) INS-1E cells were treated or not (ctrl) with IL-1β+IFNγ for 8h, chloroquine (CQ) for 3h, torin1 for 3h, or thapsigargin (Tg) for 5h. **A**) Live-cell imaging of GFP-TFEB, images are representative of 5 independent experiments. *Lower right panel*: quantitative assessment of TFEB nuclear/cytosolic localization. Scatter plot with mean ± SEM (n=20 images per condition). *P<0.05; ***P<0.001 vs. ctrl as determined by one-way ANOVA with post-hoc t-test with Tukey’s correction for multiple comparisons. **B**) Representative Western blot (*upper panel*) for TFEB and CREM-1 and quantification (*lower panel*) of TFEB over CREM-1 levels in nuclear extracts from INS-1E cells treated or not (ctrl) with IL-1β+IFNγ for 8h, or chloroquine (CQ) for 3h. Scatter plot with mean ± SEM of 5 independent experiments. *P<0.05; **P<0.01 vs. ctrl as determined by one-way ANOVA with post-hoc t-test with Tukey’s correction for multiple comparisons. **C**) Quantitative RT-PCR analyses of indicated transcript over L27 mRNA levels in INS-1E cells treated for 15h with IL-1β+IFNγ. Data are expressed as fold-changes compared to control set-up at 1 (red dotted line). Scatter plot with mean ± SEM of 5 independent experiments. *P<0.05 vs. ctrl by paired bilateral Student’s t-test.
