# Supplementary figures and images for: Dysfunctional autophagy following exposure to pro-inflammatory cytokines contributes to pancreatic β-cell apoptosis
Source: Cell Death Dis. 2018 Jan 24;9(2):96. doi: 10.1038/s41419-017-0121-5 (PMC5833699; doi:10.1038/s41419-017-0121-5)

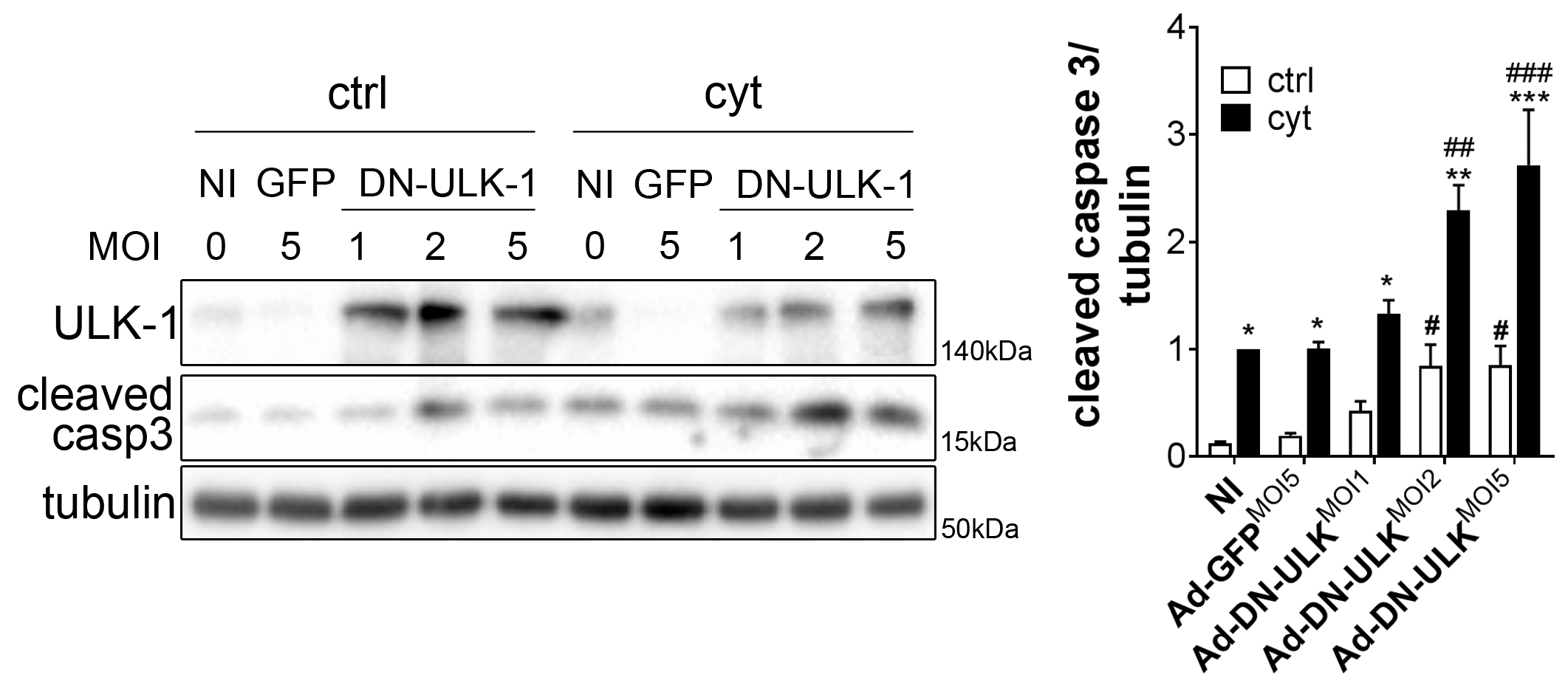

Supplement: Supplementary file 2 — Fig S1 [file 41419_2017_121_MOESM2_ESM.tif]

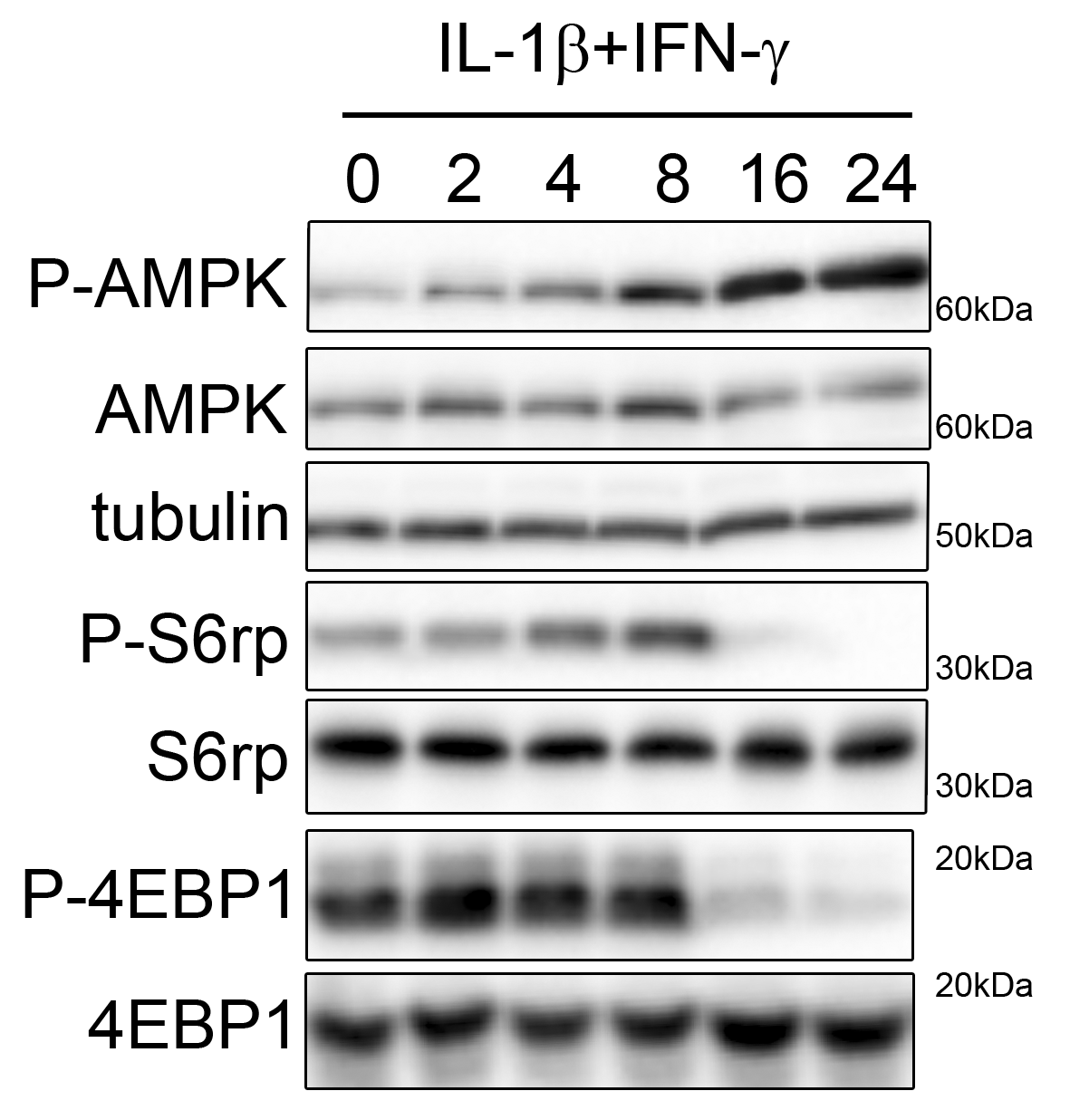

Supplement: Supplementary file 3 — Fig S2 [file 41419_2017_121_MOESM3_ESM.tif]

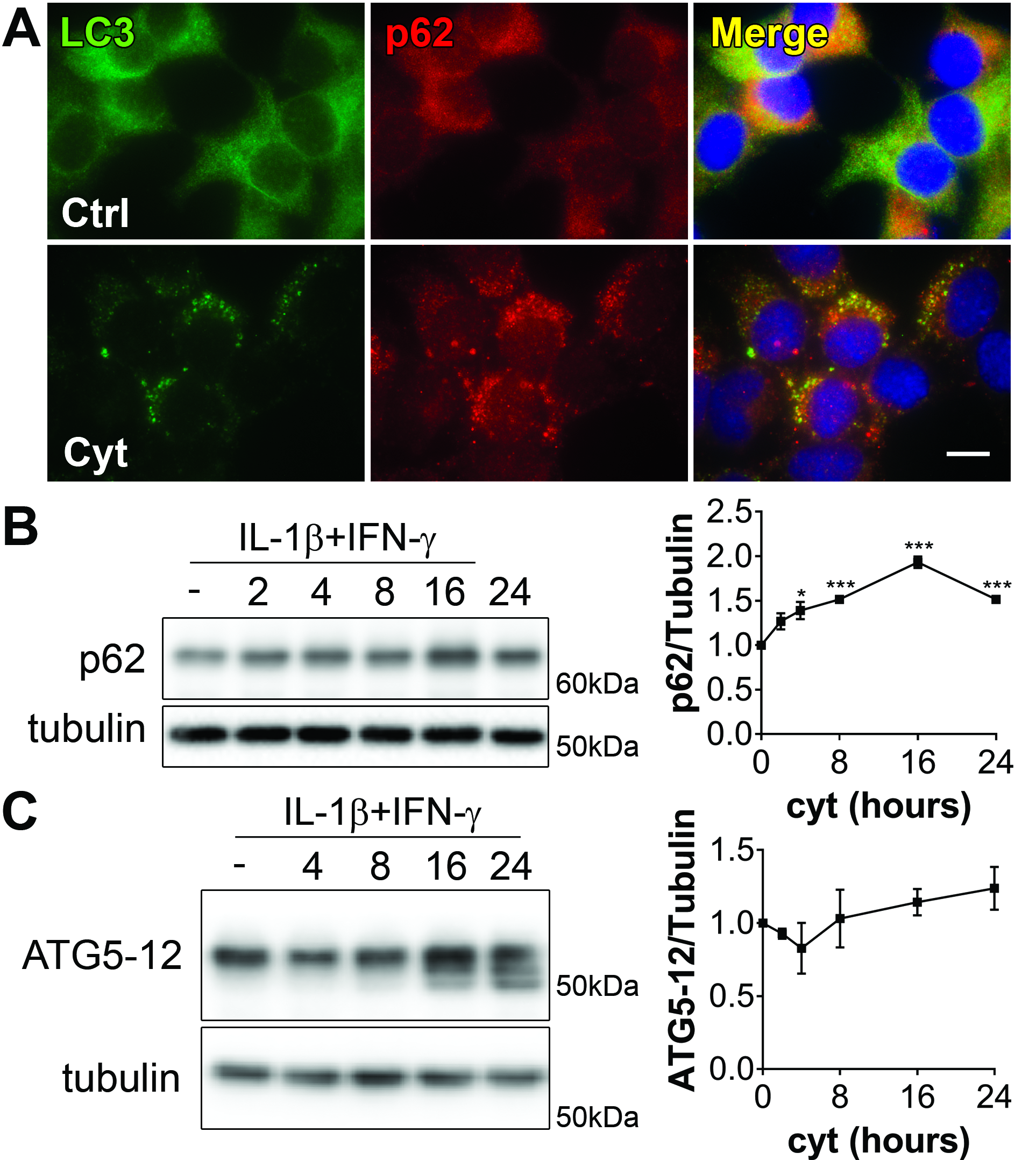

Supplement: Supplementary file 4 — Fig S3 [file 41419_2017_121_MOESM4_ESM.tif]

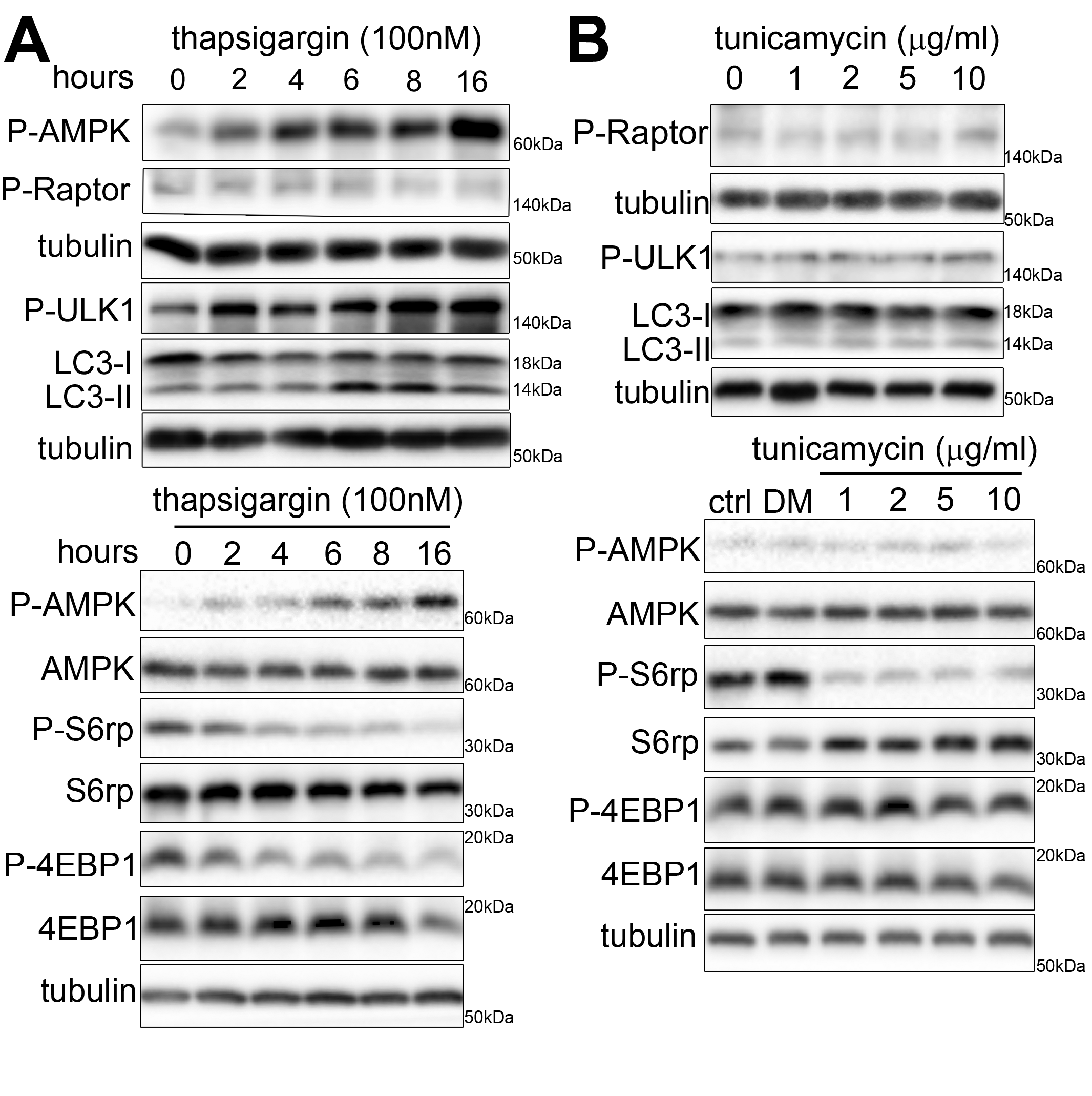

Supplement: Supplementary file 5 — Fig S4 [file 41419_2017_121_MOESM5_ESM.tif]

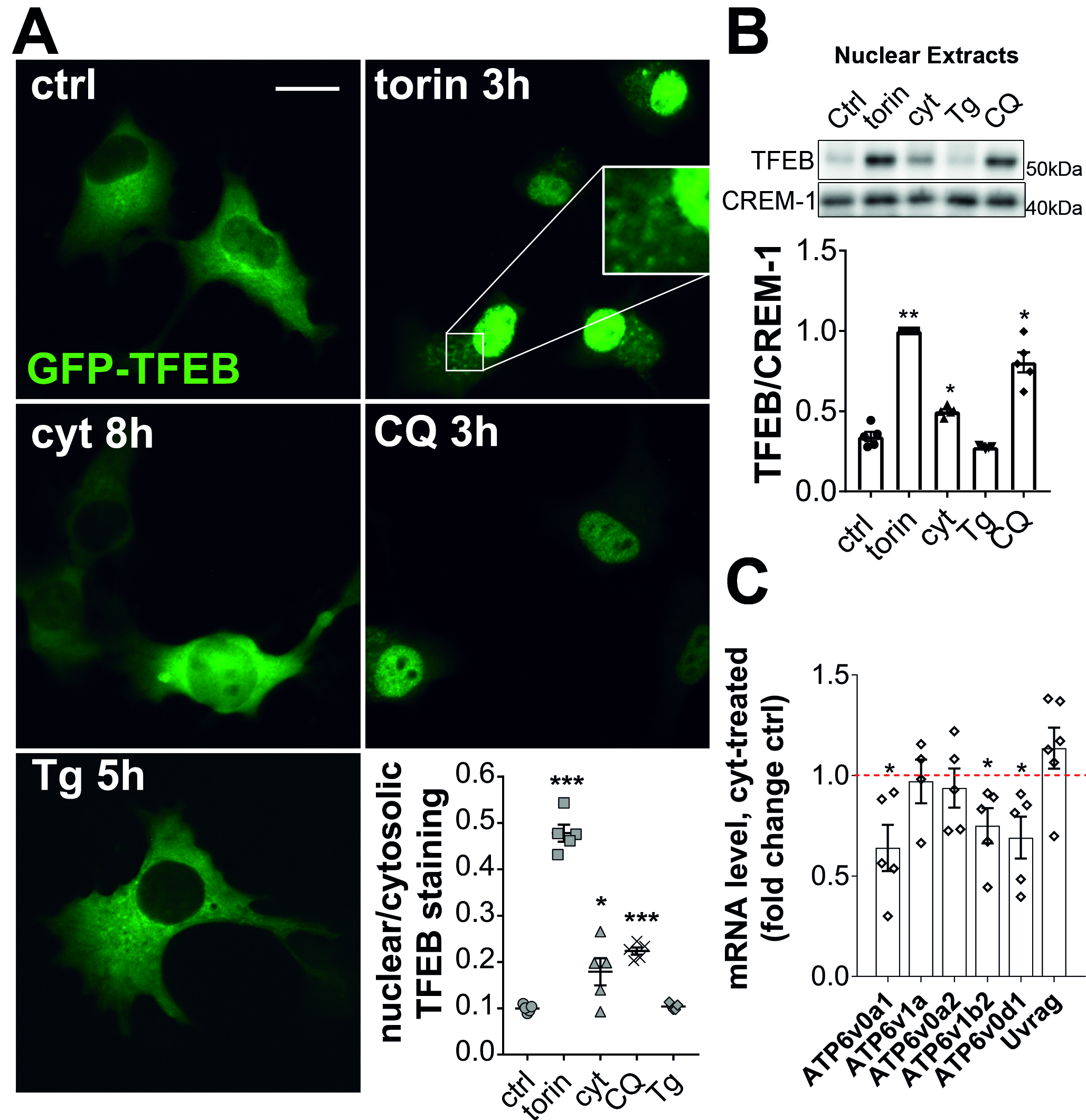

Supplement: Supplementary file 6 — Fig S5 [file 41419_2017_121_MOESM6_ESM.tif]
