# Supplementary material for: Dysfunctional autophagy following exposure to pro-inflammatory cytokines contributes to pancreatic β-cell apoptosis
Source: Cell Death Dis. 2018 Jan 24;9(2):96. doi: 10.1038/s41419-017-0121-5 (PMC5833699; doi:10.1038/s41419-017-0121-5)
Supplement: Supplementary file 7 — supplemental tables [file 41419_2017_121_MOESM7_ESM.doc]

**Electronic supplementary material**

**ESM Table 1.** Primer sequences for quantitative RT-PCR

| **Gene** | **Forward (5′–3′)** | **Reverse (5′–3′)** |
| --- | --- | --- |
| Rat Atp6v0a1 | TCAGAGGACGCAGAAGAGCCTA | GTGTTGGAGATGCAGCCCAA |
| Rat Atp6v0a2 | CCGAGACCTCAACCAAAATGT | TGGTGATTTCCTGCACCAG |
| Rat Atp6v1a | ACGAGGAAACTTCTGGTGTCTC | TGCCAGGACCAAGTTCTACC |
| Rat Atp6v1b2 | CAGCCTCGTCTCACCTACAA | GTGCCATCTGGTAATGTCAAGTG |
| Rat Atp6v0d1 | ATGCCATTCTGGTGGACACG | TCCAGATAAGCCTTGTAAAGCG |
| Rat UVRAG | AACGATGGCTACTACGGTGC | ATGAAGCCGCAGCAAAGAAA |
| RpL27 | GATCCAAGATCAAGTCCTTTGTG | CTGGGTCTCTGAACACATCCT |

**ESM Table 2.** Antibodies for Western blotting and immunofluorescence

| **Protein** | **Cat** | **Provider** |
| --- | --- | --- |
| P-4EBP-1 | 9455 | Cell Signaling Technology |
| 4-EBP-1 | 9452 | Cell Signaling Technology |
| AMPK-α | 2532 | Cell Signaling Technology |
| P-AMPK-α (Thr172) | 2535 | Cell Signaling Technology |
| CHOP | SC-575 | Santa Cruz |
| Cleaved Caspase 3 | 9661 | Cell Signaling Technology |
| CREM-1 | Sc-440 | Santa Cruz |
| P-DRP-1 (Ser616) | 3455 | Cell Signaling Technology |
| DRP-1 (DLP1) | 611112 | BD-Bioscience |
| LC3B | 3868 | Cell Signaling Technology |
| Mitofusin-2 | 9482 | Cell Signaling Technology |
| Parkin | 4411 | Cell Signaling Technology |
| P62 | GP62-C | Progen |
| P-Raptor (Ser792) | 2083 | Cell Signaling Technology |
| P-S6 Ribosomal Protein (Ser235/236) | 4858 | Cell Signaling Technology |
| S6RP | 2217 | Cell Signaling Technology |
| TFEB | A303 | Bethyl Laboratories |
| α-Tubulin | T5168 | Sigma-Aldricht |
| P-ULK-1 (Ser555) | 5869 | Cell Signaling Technology |
| ULK-1 | 8054 | Cell Signaling Technology |
| Anti-Rabbit IgG, HRP conjugate | 31460 | Thermo Fisher Scientific |
| Anti-Mouse IgG, HRP conjugate | 115-035-146 | Jackson ImmunoResearch Inc |
| Anti-Guinea pig IgG, HRP conjugate | Sc-2438 | Santa Cruz |
